# Adaptivity in Bayesian Inverse Finite Element Problems: Learning and Simultaneous Control of Discretisation and Sampling Errors

**DOI:** 10.3390/ma12040642

**Published:** 2019-02-20

**Authors:** Pierre Kerfriden, Abhishek Kundu, Susanne Claus

**Affiliations:** 1 School of Engineering, Cardiff University, The Parade, Cardiff CF243AA, UK; KunduA2@cardiff.ac.uk (A.K.); ClausS2@cardiff.ac.uk (S.C.); 2 MINES ParisTech, PSL University, Centre des Matériaux, BP87 91003 Evry, France; 3 Department of Computer Science, University of Copenhagen, Universitetsparken 1, 2100 Copenhagen, Denmark

**Keywords:** finite element inverse problems, Bayesian statistics, data-driven modelling, error estimation, MCMC, machine learning

## Abstract

The local size of computational grids used in partial differential equation (PDE)-based probabilistic inverse problems can have a tremendous impact on the numerical results. As a consequence, numerical model identification procedures used in structural or material engineering may yield erroneous, mesh-dependent result. In this work, we attempt to connect the field of adaptive methods for deterministic and forward probabilistic finite-element (FE) simulations and the field of FE-based Bayesian inference. In particular, our target setting is that of exact inference, whereby complex posterior distributions are to be sampled using advanced Markov Chain Monte Carlo (MCMC) algorithms. Our proposal is for the mesh refinement to be performed in a goal-oriented manner. We assume that we are interested in a finite subset of quantities of interest (QoI) such as a combination of latent uncertain parameters and/or quantities to be drawn from the posterior predictive distribution. Next, we evaluate the quality of an approximate inversion with respect to these quantities. This is done by running two chains in parallel: (i) the approximate chain and (ii) an enhanced chain whereby the approximate likelihood function is corrected using an efficient deterministic error estimate of the error introduced by the spatial discretisation of the PDE of interest. One particularly interesting feature of the proposed approach is that no user-defined tolerance is required for the quality of the QoIs, as opposed to the deterministic error estimation setting. This is because our trust in the model, and therefore a good measure for our requirement in terms of accuracy, is fully encoded in the prior. We merely need to ensure that the finite element approximation does not impact the posterior distributions of QoIs by a prohibitively large amount. We will also propose a technique to control the error introduced by the MCMC sampler, and demonstrate the validity of the combined mesh and algorithmic quality control strategy.

## 1. Introduction

The Bayesian statistical framework has been used extensively in the problem of system identification [1] or model updating based on experimental test data [2,3]. The objective of using inverse problems to learn or calibrate model or latent parameters, including model error terms, lies in the fact that underlying parametrised computational models are uncertain, or erroneous [4]. The experimental data when assimilated into the model, using the Bayesian inference framework, is expected to provide joint estimates of the model parameters conditional on the data and compensate for uncertainty/bias in the model predictions. However, operating only in the field of model parameters is problematic for a number of reasons. Firstly, the underlying computational models for any practical application is expensive. As a result working with a very high resolution numerical model at every stage is prohibitively expensive. Secondly, when using surrogate models in the parameter space in conjunction with a simulator, adaptive enrichment of the response surface does not take adaptive mesh refinement within its purview. This is not optimal since ensuring that the model error in the energy norm is bounded in the parameter space requires a uniformly high resolution and increases the associated computational overhead. Lastly the advanced adaptive mesh refinement techniques are rarely used in conjunction with parametric learning using Bayesian inference. There is significant room for improvement in this regard since a simultaneous control of both statistical and discretisation error would lead to substantially improved predictive numerical models both in terms of accuracy and computational efficiency.

At the root or our methodology is the estimation of errors due to the finite element approximation of the partial differential equation (PDE) of interest. Classically, spatial resolution of finite element models can be adaptively refined (also known as local *h*-refinement) based on a posteriori error estimation techniques [5,6,7], combined with re-meshing strategies. Within this, methods focussing on errors estimated in terms of specific quantities of interest (QoI), rather than the classical energy norm, constitute the goal-oriented adaptivity scheme [8,9,10,11,12], which is of particular interest in the present study. Numerical studies have shown that these give better convergence in the local features of the solution compared to traditional approaches.

Integrating model reduction techniques with finite element model updating techniques has received some attention in recent years [13], where the motivation is to use Bayesian model updating framework with an adaptive scheme of enriching the surrogate response surface. Multistage Bayesian inverse problems are quite important in this respect [14,15] and have important applications for system identification of vibrating systems. The prediction error is an important parameter to be calibrated in such cases, as the authors point out. But the improvement in model predictions, if solely focussed on obtaining the posterior probabilistic parameter estimates or for adaptive enrichment of the response surface in the parameter space, without considering simultaneous enhancement of the resolution of the numerical simulator would be unsatisfactory both from the standpoints of computational accuracy and efficiency.

The forward problem of uncertainty propagation has been investigated extensively for the solution of stochastically parametrised partial differential equations. These range from efficient stochastic Galerkin methods using polynomial chaos basis functions [16,17,18,19], stochastic collocation techniques [20,21], Monte-Carlo sampling based methods (and its various improvements) [22,23,24] and other deterministic sampling methods [25,26,27]. The main challenge is to obtain a good approximation of the lower order statistical moments of the state vector or specific quantities of interest. On the other hand, advances in the resolution of stochastic inverse problems has become a very active topic in engineering and mathematical research (see e.g., [28]). Scalable Bayesian inversion algorithms for large-scale problems have been investigated [29] and as well as Bayesian inversion to probabilistic robust optimization under uncertainty [30]. The use of adaptive sparse-grid surrogates unified with Bayesian inversion for posterior density estimates of the model or design has also been studied parameters [3,31,32]. This research focuses mostly on the definition of surrogates of the numerical model and having adaptive methods to control the statistical sampling error in the definition of the response surface. Lately there has been some research [33] that defines adaptivity as the local enrichment of the surrogate model and uses error estimation to bound this error, with no mention of spatial discretisation. However, coupling engineering uncertainty quantification (UQ) with an adaptive scheme for goal-oriented finite element model refinement remains a sparsely studied domain and presents significant challenges. It is so both from the problem formulation perspective, owing to the choice of appropriate candidate estimates based on which adaptive model enrichment can be performed, as well as incorporating it into the general formulation of Bayesian inversion.

The main focus of the paper is to develop a robust methodology for the simultaneous control of errors from multiple sources—the goal-oriented finite element error and the uncertainty-driven statistical error—in a Bayesian identification framework for identification system parameters conditional on data (experimental or otherwise). The novel algorithmic approach proposed in this paper consists in running two Markov Chain Monte Carlo algorithms simultaneously to sample the posterior densities of the quantities of interest (component-wise MCMC). The first chain utilises the current finite element model, whilst the second chain runs with a corrected likelihood function that takes into account the discretisation error. The latter quantity may be constructed by making use of a posteriori finite element error estimates available in the literature. At any time during the sampling process, two empirical densities are available and may be compared to evaluate the effect of the discretisation error onto posterior densities. The second building block of our algorithmic methodology allows us to determine whether enough samples have been drawn by the component-wise MCMC. Using multiple parallel chains combined with bootstrap-based estimates of sampling errors, we automatically stop the MCMC algorithms when either (i) the required level of accuracy is achieved or (ii) we have generated enough statistical confidence in the fact that the discretisation error is too large for our purpose, and therefore that mesh refinement is necessary. While any available error estimate of the discretisation error may be used within the general strategy outlined above (a goal-oriented residual or recovery-based error estimate, for instance [8,9,11,34]), we choose instead to construct this estimate via a dedicated machine learning approach. This interesting feature, inspired by previous work in data-driven error modelling [35,36,37,38], will be briefly outlined in the paper.

The paper is organised as follows. Section 2 introduces the Bayesian inverse problem based on a finite element model of a parametrised vibrating structure. This section also includes discussions on discretisation error. Some numerical examples are presented in Section 3, which aims to demonstrate the convergence of the joint posterior distributions on the model parameters through successive stages of mesh refinement. Section 4 discusses the total error as a combination of statistical and discretisation error that results from the MCMC algorithm used for sampling from posterior distributions. Section 5 gives the methodology for robust, simultaneous control of all error sources within the adaptive inverse problem solver using a component-wise MCMC algorithm in conjunction with bootstrap-aggregated regression model for model parameters. Numerical examples are presented and discussed in Section 6 to demonstrate the capabilities of the proposed methodology.

## 2. Finite Element Bayesian Inverse Problems

Although the methodology proposed in this paper is general, we will apply it to the verification of popular inverse finite element procedures used to monitor the integrity of structures during service. We will assume that the structure can be modelled as an elastic body, and that potential structural damage can be modelled by the evolution of a field of elastic constants characterised by a finite number of uncertain parameters. Assuming that the resonance frequencies of the structure have been measured physically, we attempt to identify this set of mathematical parameters, through the solution of an inverse problem. Significant deviations from the parameter values corresponding to an undamaged structural state may reveal structural failure. This is a typical condition monitoring method used for non-destructive structural testing.

**Figure 1:**
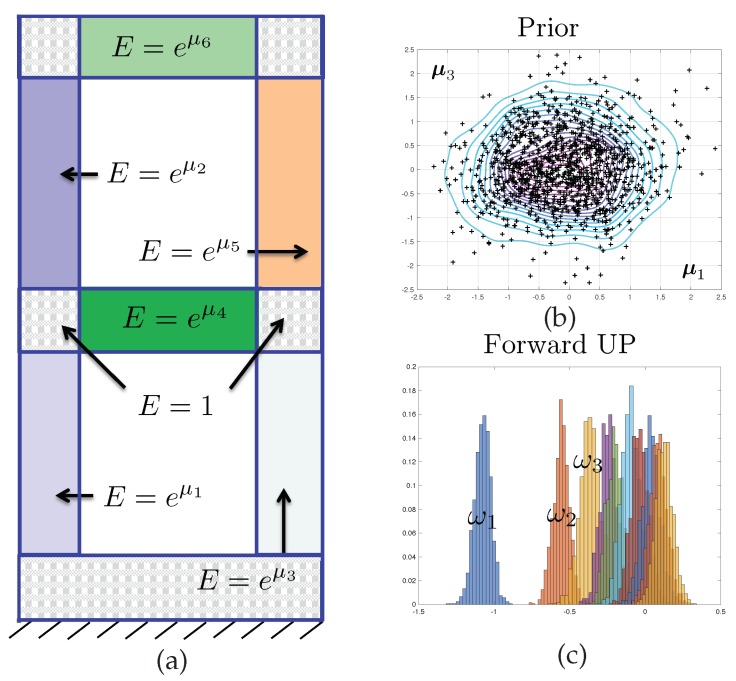
(**a**) Structure undergoing structural vibrations. The grey area have a known, deterministic Young modulus. This property is piecewise constant in the coloured areas, and Gaussian distributed. (**b**) The corresponding marginal probability density function for two of the 6 parameters is represented in the top-left corner. (**c**) Standard Monte-Carlo finite element process is employed to compute the distribution of the 10 first free vibration frequencies and represent the marginal distribution of each of these quantities.

In order to clarify the proposed study, we will assume that we have measured the $n_{d}$ first dynamic eigenfrequencies $d={\left(\begin{array}{ccc} \ln(\omega_{1}) & ... & \ln(\omega_{n_{d}}) \end{array}\right)}^{T}$ of the structure represented in Figure 1. The purpose of the model inversion is to identify the values of $n_{\mu }$ parameters $\mu ={\left(\begin{array}{ccc} \mu_{1} & ... & \mu_{n_{\mu }} \end{array}\right)}^{T}$ of the structural dynamics model, here the log of the elastic modulii of the subdomains represented in Figure 1. We will also aim to predict the remaining $n_{p}-n_{d}$ frequencies $p={\left(\begin{array}{ccc} \ln(\omega_{n_{d}+1}) & ... & \ln(\omega_{n_{d}+n_{p}}) \end{array}\right)}^{T}$. The relationships μ→d(μ) and μ→p(μ), i.e., the computational model, are defined implicitly through the evaluation of a standard finite element model of the steady-state, undamped structural vibrations.

### 2.1. Bayesian Inverse Problem

We assume that the quantities d that are measured experimentally are described by the mathematical model, up to an error, which is modelled in a probabilistic manner. Following standard Bayesian procedures, this error is modelled as a white Gaussian noise,

(1) $$d=d_{m}+\epsilon \;,\;\epsilon ∼N\left(0,\Sigma_{n}^{-1}\right)$$

with $\Sigma_{0}$ a positive definite covariance matrix. $A_{d}:\mu \to d_{m}:=A_{d}\left(\mu \right)$ is the mathematical prediction that corresponds to the experimental observation. Model $A_{d}$ is a deterministic function of $n_{\mu }$ uncertain model parameters that we organise in a $n_{\mu }$-dimensional vector μ. Bayesian inversion requires to associate a prior probability density with the model parameters μ. This prior probability density, denoted by $\pi_{\mu }\left(\mu \right)$ in the following, encodes the knowledge that we possess about μ before making any physical observations.

Seen from a different point of view, the probabilistic inversion setting amounts to the definition of a joint probability distribution for μ and d:

(2) $$\pi_{\mu ,d}\left(\mu ,d\right)=\pi_{d|\mu }\left(\mu ,d\right)\;\pi_{\mu }\left(\mu \right)$$

The formal expression of likelihood function $L\left(\mu ;d\right):=\pi_{d|\mu }\left(\mu ,d\right)$ is a direct consequence of assumption (1), namely

(3) $$L\left(\mu ;d\right)=\frac{1}{\sqrt{{\left(2\pi \right)}^{n_{d}}\left|\Sigma_{n}\right|}}e^{-\frac{1}{2}\left({\left(d-A_{d}\left(\mu \right)\right)}^{T}\Sigma_{n}^{-1}\left(d-A_{d}\left(\mu \right)\right)\right)}$$

It is now possible to formally proceed to the inversion itself by conditioning the joint distribution to actually observed quantities. Applying Bayes’ formula, the posterior probability density of the model parameters is

(4) $$\pi_{\mu |d}\left(\mu ;d\right)=\frac{L\left(\mu ;d\right)\;\pi_{\mu }\left(\mu \right)}{\pi_{d}\left(d\right)}\;$$

where $\pi_{d}\left(d\right)$ is a normalising constant, whose computation requires a usually intractable integration. Bayes formula provides us with an updated knowledge about the uncertain part of our mathematical model. It is now possible to predict unobserved quantities i.e.,

(5) $$p=A_{p}\left(\mu \right)\;,\;\;\mu ∼\pi_{\mu }|d\left(\mu ;d\right)\;$$

by formally propagating the posterior uncertainty through model $A_{p}:\mu \to p_{m}:=A_{p}\left(\mu \right)$. The posterior predictive probability density of p will be denoted by symbol $\pi_{p|d}\left(\mu ;d\right)$.

### 2.2. Finite Element Modelling of Inverse Structural Vibration Problems

#### 2.2.1. Direct Finite element Procedure for Frequency-Domain Vibrations

The numerical model

(6) $$\mu \to A\left(\mu \right):=\left(\begin{array}{c} A_{d}\left(\mu \right)\\ A_{p}\left(\mu \right) \end{array}\right)$$

is implicitly defined through the solution of a continuum mechanics problem. The components of $d_{m}$ and p are the logarithm of the eigenvalues corresponding to the following parametrised eigenvalue problem: *Find $u\in H^{1}\left(\Omega \right)$ and $\omega \in R^{+}$ such that $\forall v\in H^{1}\left(\Omega \right)$*

(7) $$\int_{\Omega }\;\nabla_{s}u:D\left(x;\mu \right):\nabla_{s}v\;d\Omega -\omega^{2}\int_{\Omega }\rho \;u\cdot v\;d\Omega =0\;$$

In the previous variational statement, Ω is the domain occupied by the structure of interest, $H^{1}\left(\Omega \right)$ is the space of functions defined over Ω, with values in $R^{2}$, that are zero on part $\partial_{u}\Omega$ of the boundary of the domain, and whose derivatives up to order one are square integrable. $\nabla_{s}$ denotes the symmetric part of the gradient operator. D is the fourth order Hooke tensor and ρ is the mass density of the solid material. The problem possesses an infinite number of solutions (ω,u) called free vibration modes. We order the free vibration frequencies in increasing order $\omega_{1}<\omega_{2}<\;...\;<\omega_{n_{d}+n_{p}}$. The free vibration modes are functions of uncertain material parameters μ through the definition of the Hooke tensor. Specifically,

(8) $$D\left(x;\mu \right):\nabla_{s}\;.\;=\lambda \left(x;\mu \right)\mathrm{Tr}\left(\nabla_{s}\;.\;\right)\;I+2\;G\left(x;\mu \right)\left(\nabla_{s}\;.\;\right)\;$$

with the parametrised Lamé constants

(9) $$\lambda \left(x;\mu \right)=\frac{0.3\times E(x;\mu )}{2(1+0.3)(1-2\times 0.3)}\;\mathrm{and}\;G\left(x;\mu \right)=\frac{E(x;\mu )}{2(1+0.3)}$$

Solving the continuum mechanics model is equivalent to evaluating the mapping

(10) $$\mu \to \left(\begin{array}{c} A_{d}\left(\mu \right)\\ A_{p}\left(\mu \right) \end{array}\right)=\left(\begin{array}{c} \ln{\left(\begin{array}{ccc} \omega_{1}\left(\mu \right) & ... & \omega_{n_{d}}\left(\mu \right) \end{array}\right)}^{T}\\ \ln{\left(\begin{array}{ccc} \omega_{n_{d}+1}\left(\mu \right) & ... & \omega_{n_{d}+n_{p}}\left(\mu \right) \end{array}\right)}^{T} \end{array}\right).$$

There is, in general, no analytical solution to the continuous vibration problem, and a standard way to obtain approximate solutions is to substitute a finite element space $U^{h}\left(\Omega \right)$ for infinite dimensional search space $H^{1}\left(\Omega \right)⊃U^{h}\left(\Omega \right)$ [39]. Too coarse a finite element discretisation may result in poorly predictive results, while too fine a mesh will lead to numerically intractable results, or, in any case, to a waste of computing resources.

#### 2.2.2. Finite Element Approximation of the Bayesian Inverse Problem

Whilst using the continuum mechanics model exactly would deliver the posterior density $\pi_{\mu |d}\left(\mu ;d\right)$ as solution of the Bayesian inverse problem, we now have an approximate posterior density

(11) $${\bar{\pi }}_{\mu }|d\left(\mu ;d\right)=\frac{\bar{L}\left(\mu ;d\right)\;\pi_{\mu }\left(\mu \right)}{\bar{\pi }\left(d\right)}$$

where the finite element likelihood $\bar{L}$ is obtained by substituting finite element mapping ${\bar{A}}_{d}$ for $A_{d}$ in Equation (3). Similarly, the approximate posterior density of p is denoted by ${\bar{\pi }}_{p|d}\left(\mu ;d\right)$ and obtained by evaluating finite element mapping ${\bar{A}}_{p}$ instead of $A_{p}$ when propagating the posterior uncertainties forward.

#### 2.2.3. Discretisation Error

The finite element error is the mismatch between ${\bar{\pi }}_{\mu |d}\left(\mu ;d\right)$ and $\pi_{\mu |d}\left(\mu ;d\right)$ on the one hand, and ${\bar{\pi }}_{p|d}\left(\mu ;d\right)$ and $\pi_{p|d}\left(\mu ;d\right)$ on the other hand. Various measures can be used to quantify this mismatch, amongst which the Hellinger distance, defined by

(12) $$D_{h}\left(\pi_{\mu |d}\left(\mu \right),{\bar{\pi }}_{\mu |d}\left(\mu \right)\right)={∥\sqrt{\pi_{\mu |d}\left(\mu \right)}-\sqrt{{\bar{\pi }}_{\mu |d}\left(\mu \right)}∥}_{2}$$

the Kullback-Leibler divergence, the total variation distance and the Kolmogorov-Smirnov (KS) distance, defined by

(13) $$D_{\mathrm{ks}}\left(\pi_{\mu |d}\left(\mu \right),{\bar{\pi }}_{\mu |d}\left(\mu \right)\right)=\underset{\mu }{\sup}\left(\left|(\Pi_{\mu |d}\left(\mu \right)-{\bar{\Pi }}_{\mu |d}\left(\mu \right))\right|\right)$$

where the capital symbols $\Pi_{.}$ denotes the cumulative probability density corresponding to $\pi_{.}$, and ${\bar{\Pi }}_{.}$ is the finite element approximation of Π. This contribution will make use of the latter measure, in a one-dimensional setting (i.e., it will be applied to control the accuracy of the posterior density of one of the elements of μ or one of the elements of p). The attractiveness of the Kolmogorov–Smirnov distance is its straightforward application in the context of Monte-Carlo procedures, where only empirical densities are available, and its closeness to confidence intervals (CIs), which makes its values relatively easy to interpret within the context of a posteriori error estimation. Notice that the $D_{\mathrm{ks}}$ is lower bounded by 0 (identical density functions) and upper bounded by 1 (non-overlapping support for the probability density functions).

## 3. Numerical Examples—Part I: Effect of Discretisation Errors Onto Posterior Densities

This section introduces the numerical examples that will be investigated in this paper, and aims to provide a first qualitative understanding of the effect of mesh refinement onto the quality of posterior probability densities.

### 3.1. Forward Stochastic Model

The stochastic field of Young’s modulus that will be used to exemplify the error control approach proposed in this paper is defined via a decomposition of domain Ω into $n_{\mu }+1=7$ non-overlapping subdomains ${\left\{\Omega_{i}\right\}}_{〚1\;n_{\mu }+1〛}$ such that $\underset{i\in 〚1\;n_{\mu }+1〛}{⋃}\Omega_{i}=\Omega$ and $\Omega_{i}\cap \Omega_{j}=\left\{\;\right\}$ for i≠j. The domain decomposition is represented in Figure 1. Then, the proposed model is such that

(14) $$E\left(x;\mu \right)=e^{\mu_{i}}\;\mathrm{if}\;x\in \Omega_{i}\;\mathrm{and}\;i\neq 1,\;E\left(x;\mu \right)=1\;\mathrm{otherwise}.$$

Hence, the scalar parameters contained in vector $\mu \in R^{n_{\mu }}$ are the logarithms of the Young’s modulii corresponding to each of the subdomains.

The prior density is a multivariate Gaussian, and is given by equation

(15) $$\pi_{\mu }\left(\mu \right)=\frac{1}{\sqrt{{\left(2\pi \right)}^{n_{\mu }}\left|\Sigma_{0}\right|}}e^{-\frac{1}{2}\left({\left(\mu -\mu_{0}\right)}^{T}\Sigma_{0}^{-1}\left(\mu -\mu_{0}\right)\right)}$$

The prior mean $\mu_{0}$ is the null vector, and the prior variance $\Sigma_{0}=\sigma_{0}^{2}\;I_{d}$, where $I_{d}\in R^{n_{\mu }}\times R^{n_{\mu }}$ is the identity matrix, is diagonal and isotropic. The prior density is represented in dimensions $\left(\mu_{1},\mu_{3}\right)$ in Figure 1.

Finally, we model the error ϵ as a zero-mean multivariate Gaussian (consistently with what was described in previous section), with independent components and isotropic variance, i.e., $\Sigma_{n}=\sigma_{n}^{2}\;I_{d}$.

### 3.2. Computational Meshes

**Figure 2:**
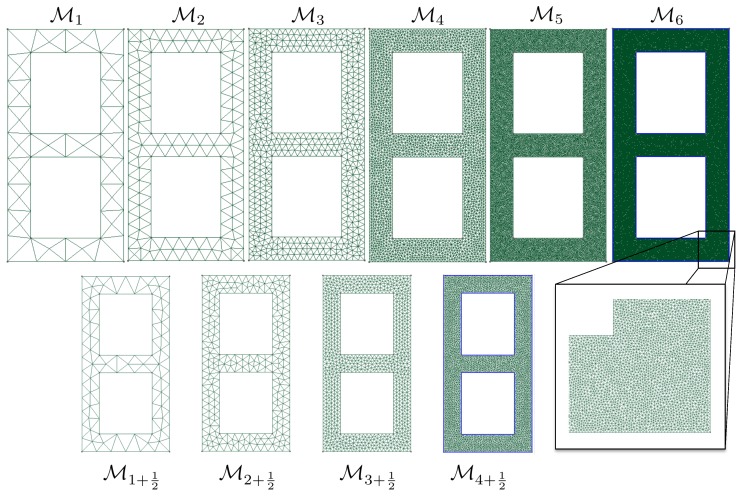
Hierarchy of computational meshes used in the study. We aim at selecting the coarsest mesh that delivers the targeted distributions up to a user-defined numerical accuracy. The second line of meshes is only used for error estimation purposes.

The evaluation of the likelihood function appearing in solution (11) of the Bayesian inverse problem requires solving the continuum mechanics problem using the finite element method. In this example, we use a sequence of meshes ${\left\{M_{i}\right\}}_{i\in 〚\;\;n_{m}〛}$ associated with a monotonically increasing number of degrees of freedom. These meshes are represented in Figure 2. Although the sequence of meshes is not strictly hierarchical, we see that the typically uniform element size is divided by ≈2 when moving from mesh $M_{i}$ to mesh $M_{i+1}$. The intermediate meshes ${\left\{M_{i+\frac{1}{2}}\right\}}_{i\in 〚1\;{\tilde{n}}_{m}〛}$ represented in Figure 2 will be used later on.

### 3.3. Inverse Problems and First Results

Two tests will now be investigated:

- Test 1(weakly informative data): only the first eigenvalue is measured, i.e., d is scalar. This can be interpreted as a task of model updating, where new data is used to update an existing knowledge.
- Test 2 (strongly informative data): the first three eigenvalues of the structure are measured. This can be interpreted as an inverse problem, where rich information is used to identify all the unknown of the model, and the probabilistic setting acts as a regulariser.

**Figure 3:**
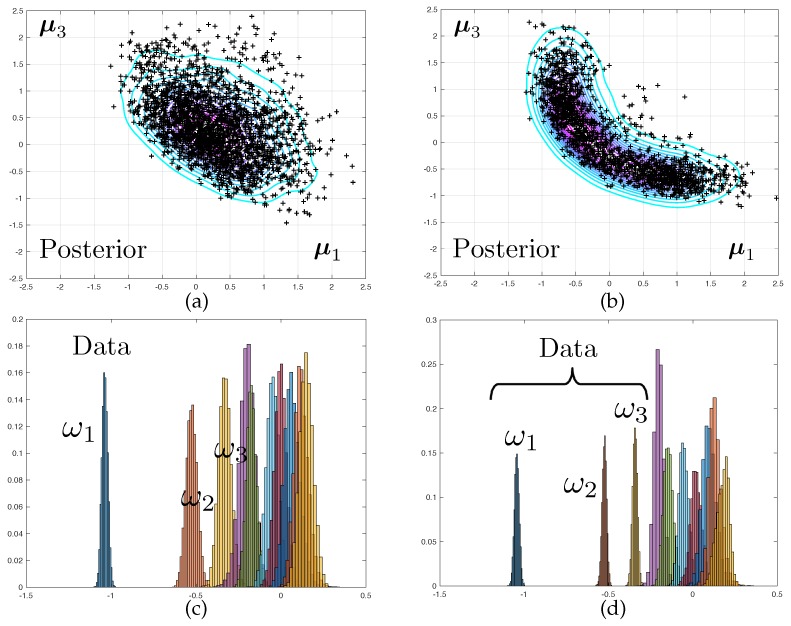
(**a**) Joint posterior probability distribution for two of the 6 unknown elastic modulii and (**c**) marginal posterior predictive distributions of the first 10 free vibration frequencies. The data set consists of a noisy measurement of the first frequency only, resulting in fat posterior densities. (**b**) Posterior distribution of the two unknown elastic modulii when the first 3 vibration frequencies are used as dataset, which results as in much thinner posterior densities. (**d**) Corresponding posterior predictive densities.

The two corresponding marginal posterior density ${\hat{\pi }}_{\mu_{1},\mu_{3}|d}$ obtained when using mesh $M_{3}$ are represented in Figure 3. Samples from this density are obtained by a Monte-Carlo sampler, as presented in next section. A Kernel Density Estimate (KDE) is used as a smoother for illustration purposes only. Notice that for the predictive posterior densities, the histograms correspond to the marginal densities of each individual eigenvalue, which explains their overlap.

It is interesting to notice that the posterior densities observed in Test 2 are much sharper than that of Test 1, owing to the quality of the data. The symmetry in the results are a consequence of structural and probabilistic symmetries (see Figure 1 and the definition of the prior probability density).

Synthetic data for this problem is generated by computing the average of the spectra delivered by meshes $M_{2}$ and $M_{2+\frac{1}{2}}$, for a reference parameter vector $\mu_{d}\neq \mu_{0}$ but situated in the vicinity of the prior mode. The model averaging, together with selecting relatively coarse finite element meshes in sequence ${\left\{M_{i}\right\}}_{i\in 〚\;\;n_{m}〛}$, is meant to circumvent the “inverse crime" problem.

### 3.4. Convergence with Mesh Refinement

**Figure 4:**
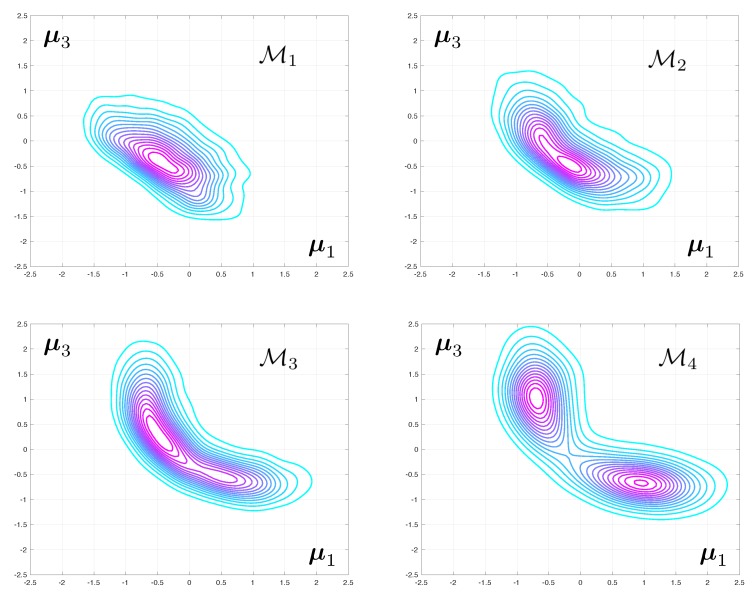
Evolution of the joint posterior density of two of the unknown elasticity parameters as the computational mesh is progressively refined from $M_{1}$ to $M_{4}$.

The posterior densities corresponding to Test 2 and to various level of mesh refinement are displayed in Figure 4. The difference between the modes of the predicted eigenvalues that are obtained with coarse and fine meshes are very large. The fact that this discrepancy increases with the mode number is to be expected as the spatial wave length of the deformations in the continuum can be shown to increase linearly with the eigenfrequency. As a consequence, a good mesh for the first range of frequencies might be unable to capture the faster spatial variations associated with higher vibration mode.

The difference between the posteriori densities corresponding to meshes $M_{3}$ and $M_{4}$ is qualitatively small. The solution of the Bayesian inverse problem converges with mesh refinement. Notice that the posterior distribution for the model parameters goes from mono-modal to multi-modal, which may prove a stumbling block when selecting an appropriate Monte-Carlo sampler.

## 4. Monte-Carlo Sampler and Combined Effect of Statistical and Discretisation Errors

### 4.1. Tempered Metropolis-Hastings Markov Chain Monte Carlo Algorithm

The approximate posterior density described by Equation (11) is an arbitrarily complex function of μ, as it depends on the *nonlinear* computational model ${\left(A_{d}{\left(\mu \right)}^{T}\;A_{p}{\left(\mu \right)}^{T}\right)}^{T}$. In particular, standard random number generators cannot be used to draw samples from this distribution. Importance Monte-Carlo procedures whereby the prior distribution is used as proposal density, will also fail. This is because the posterior density may be arbitrarily different from the prior density, resulting in unacceptably large variances of importance sampling estimates. Designing better proposal densities a priori is impossible and a successful importance sampling approach would require generating the proposal density using advanced methods such as sequential Monte-Carlo samplers.

**Figure 5:**
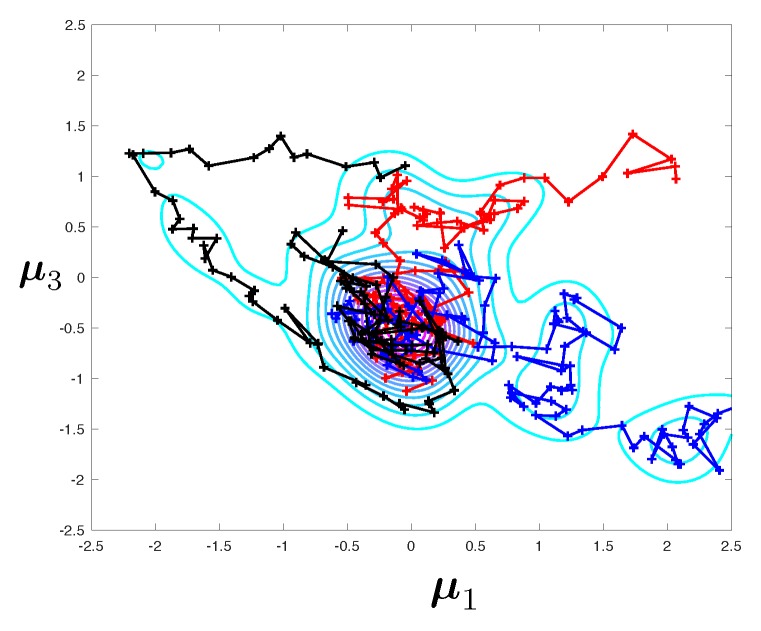
Three independent MCMC chains sampling a multivariate Gaussian distribution.

One of the simplest and generic generator of samples from posterior densities is the Metropolis-Hastings (MH) Markov Chain Monte Carlo (MCMC) algorithm (see Figure 5). The algorithm works as follows. Starting from sample $\mu_{n}$, the next sample $\mu_{n+1}$ is drawn from transitional distribution

(16) $$T_{\mu_{n+1}\leftarrow \mu_{n}}\left(\mu_{n+1};\mu_{n}\right)\propto \min\left(1,\frac{{\bar{\pi }}_{\mu |d}\left(\mu_{n+1}\right)}{{\bar{\pi }}_{\mu |d}\left(\mu_{n}\right)}\right)N\left(\mu_{n+1};\mu_{n},\tilde{\Sigma }\right)$$

This is done by first drawing a random move from $\mu_{k}$ using the multivariate Gaussian (any proposal can be used, but the expressions exposed in this section are only valid for symmetric proposals), and then accepting or rejecting the move in order to account for the first term of the transition. Typically, a move ending up in a state of higher posterior density is always accepted, whilst a move ending up in a state of lower density may be accepted or not, depending on the result of a die and the ratio of posterior densities between current and proposed states.

This particular transition is designed such that the following ergodic property holds:

(17) $$\int_{\mu_{n+1}}T_{\mu_{n+1}\leftarrow \mu_{n}}\left(\mu_{n+1};\mu_{n}\right)\;d\mu_{n}={\bar{\pi }}_{\mu |d}\left(\mu_{n+1}\right)$$

As a result, under some assumptions, the Markov chain is guaranteed to have ${\bar{\pi }}_{\mu |d}$ as its stationary distribution, which means that $\mu_{n}∼{\bar{\pi }}_{\mu |d}$ as n→∞. Each sample, taken individually, is distributed according to the posterior density, if the chain is run long enough. Due to the Markov process, the samples are not independent, which makes frequentist error estimation and convergence diagnosis more difficult than in the context of traditional Monte-Carlo algorithm, where tools such as standard error and bootstrap apply without particular difficulty. Practically, a burn-in phase is first observed, whereby the chain “seeks” the regions of high probability densities (see Figure 5). Once found, samples become progressively distributed according to the target posterior density.

Notice that $\tilde{\Sigma }$ is difficult to choose a priori. Adaptive proposal MCMC have been proposed in [40,41]. Alternative solutions to choose good proposal are methods based on particle mechanics (e.g., Langevin diffusion, Hamiltonian dynamics) (see e.g., [42]). In this particular contribution, the proposal densities have been calibrated “by hand”. We use a tempered version of the MCMC algorithm, whereby we simultaneously sample multiple tempered replicates of the posterior density, with proposed state exchange between replicates that are subsequently Metropolis corrected [43,44] (see also [45] for an interesting application in the context of structural damage assessment). This relatively classical MCMC algorithm allows us to sample from; densities that are multi-modal or become multi-modal with mesh refinement (at least in the low-dimensional parametric settings that is investigated in this paper, as multi-modality in high-dimensions remains an open problem).

The call to the finite element solver is hidden in the acceptance/rejection test, which requires the evaluation of the finite element likelihood at the proposed state. Therefore, as for a standard MC algorithm, one iteration means one evaluation of the computational model.

### 4.2. Empirical Posterior Densities

Once *n* samples $\tilde{M}=\left\{\mu_{1}\;,\;\mu_{2},\;...\;,\;\mu_{n}\right\}$ have been computed (*n* larger than 10) by the MH algorithm, we discard the first 25% (burn-in) of these samples, resulting in $\tilde{n}$ samples that we hope are located in the ergodic part of the Markov chain. The resulting empirical probability density is given by

(18) $${\bar{\pi }}_{\mu |d}\left(\mu \right)\approx {\bar{\pi }}_{\mu |d}^{\left(n\right)}\left(\mu \right):=\frac{1}{\tilde{n}}\sum_{k=n-\tilde{n}+1}^{n}\delta \left(\mu -\mu_{k}\right)$$

where δ is the Dirac delta function. Accordingly, the empirical predictive posterior density is

(19) $${\bar{\pi }}_{p|d}\left(p\right)\approx {\bar{\pi }}_{p|d}^{\left(n\right)}\left(p\right):=\frac{1}{\tilde{n}}\sum_{k=n-\tilde{n}+1}^{n}\delta \left(p-A_{p}\left(\mu_{k}\right)\right)$$

The cumulative empirical predictive posterior density may be expressed as

(20) $${\bar{\Pi }}_{\mu |d=d}\left(\mu \right):=\frac{1}{\tilde{n}}\sum_{k=n-\tilde{n}+1}^{n}I\left(\mu ;\mu_{k}\leq \mu \right)$$

where I is the indicator function, and the inequality is to be understood in a component-by-component manner. A similar definition holds for the cumulative posterior density of p.

### 4.3. Total Error Measure

It is now clear that both the finite element discretisation and the Monte-Carlo approximate sampling affect the quality of the resulting posterior densities. The total error, for the marginal distribution of a single element $\mu_{i}$ of μ, reads as

(21) $$D_{\mathrm{ks}}\left(\pi_{\mu_{i}|d}\left(\mu_{i}\right),{\bar{\pi }}_{\mu_{i}|d}^{\left(n\right)}\left(\mu_{i}\right)\right)=\underset{\mu_{i}}{\sup}\left(\left|(\Pi_{\mu_{i}|d}\left(\mu_{i}\right)-{\bar{\Pi }}_{\mu_{i}|d}^{\left(n\right)}\left(\mu_{i}\right))\right|\right)$$

which can be formally decomposed as follows

(22) $$\begin{array}{ccc} D_{\mathrm{ks}}\left(\pi_{\mu_{i}|d}\left(\mu_{i}\right),{\bar{\pi }}_{\mu_{i}|d}^{\left(n\right)}\left(\mu_{i}\right)\right) & = & \underset{\mu_{i}}{\sup}\left(\left|\left(\Pi_{\mu_{i}|d}\left(\mu_{i}\right)-{\bar{\Pi }}_{\mu_{i}|d}\left(\mu_{i}\right)\right)+{\bar{\Pi }}_{\mu_{i}|d}\left(\mu_{i}\right)-{\bar{\Pi }}_{\mu_{i}|d}^{\left(n\right)}\left(\mu_{i}\right))\right|\right)\\ & \leq & D_{\mathrm{ks}}\left(\pi_{\mu_{i}|d}\left(\mu_{i}\right),{\bar{\pi }}_{\mu_{i}|d}\left(\mu_{i}\right)\right)+D_{\mathrm{ks}}\left({\bar{\pi }}_{\mu_{i}|d}\left(\mu_{i}\right),{\bar{\pi }}_{\mu_{i}|d}^{\mathrm{mc}}\left(\mu_{i}\right)\right) \end{array}$$

In the last expression, the first term is the pure finite element error, which would occur if we could run the Markov process for an infinite number of iterations, while the second term is a pure statistical error.

We also define the error of an element $p_{i}$ of p as follows:

(23) $$D_{\mathrm{ks}}\left(\pi_{p_{i}|d}\left(p_{i}\right),{\bar{\pi }}_{p_{i}|d}^{\left(n\right)}\left(\mu_{i}\right)\right)=\underset{p_{i}}{\sup}\left(\left|(\Pi_{p_{i}|d}\left(p_{i}\right)-{\bar{\Pi }}_{p_{i}|d}^{\left(n\right)}\left(p_{i}\right))\right|\right)$$

## 5. Robust, Automatised and Comprehensive Error Control

### 5.1. Simulation of the Discretisation Error

The finite element method introduces an error in computational mapping $A\left(\mu \right)={\left(\begin{array}{cc} A_{d}{\left(\mu \right)}^{T} & A_{p}{\left(\mu \right)}^{T} \end{array}\right)}^{T}$. Mapping A contains all the scalar quantities that need to be evaluated through calls to the finite element solver, namely the numerical predictions of the physical measurements d, and the posterior predictions p. We define the error in the simulated data as

(24) $$\Delta A_{d}\left(\mu \right)=A_{d}\left(\mu \right)-{\bar{A}}_{d}\left(\mu \right)$$

and the error in the posteriori predictions as

(25) $$\Delta A_{p}\left(\mu \right)=A_{p}\left(\mu \right)-{\bar{A}}_{p}\left(\mu \right)$$

For now, we assume that both these quantities can be estimated, at affordable numerical cost and in a reliable manner. Therefore, for any value of parameter μ, a corrected computational model is available, which reads as

(26) $$\left(\begin{array}{c} A_{d}\left(\mu \right)\\ A_{p}\left(\mu \right) \end{array}\right)\underset{\mathrm{very}\;\mathrm{close}\;\mathrm{to}}{\approx }\left(\begin{array}{c} {\hat{A}}_{d}\left(\mu \right)\\ {\hat{A}}_{p}\left(\mu \right) \end{array}\right):=\left(\begin{array}{c} {\bar{A}}_{d}\left(\mu \right)\\ {\bar{A}}_{p}\left(\mu \right) \end{array}\right)+\left(\begin{array}{c} {\hat{\Delta A}}_{d}\left(\mu \right)\\ {\hat{\Delta A}}_{p}\left(\mu \right) \end{array}\right)$$

where symbol $\hat{.}$ denotes computable estimates.

### 5.2. Component-Wise MCMC

It is now posible to sample the corrected posterior distribution

(27) $${\hat{\pi }}_{\mu |d}\left(\mu ;d\right)=\frac{\hat{L}\left(\mu ;d\right)\;\pi_{\mu }\left(\mu \right)}{\hat{\pi }\left(d\right)}$$

using MCMC. It should be clear that the corrected posterior distribution is simply obtained by replacing the corrected computational model (26) into the expression of the likelihood function, Equation (3). Notice that the normalising constant is affected by modifications of the computational model. This is of no practical consequence as MCMC samplers work with unnormalised densities, and the KS distance uses empirical cumulative distributions directly, without the need for smoothing or marginalisation.

We formally define a component-wise MCMC were the uncorrected and corrected computational models are sampled at the same time. This will yield an estimate of the effect of the discretisation error on posteriori densities at any stage of the Markov process, which, in turn, will allow us to develop an early-stopping methodology. The Component-wise MCMC iteration proceeds as follows, given a current sample $\left({\bar{\mu }}_{n},{\hat{\mu }}_{n}\right)$ of the uncorrected/corrected finite element posteriori densities,

- Draw $\left({\bar{\mu }}_{n+1},{\hat{\mu }}_{n+1}\right)$ such that
  (28) $${\bar{\mu }}_{n+1}∼N\left(\;.\;;{\bar{\mu }}_{n},\tilde{\Sigma }\right)\;\mathrm{and}\;{\hat{\mu }}_{n+1}∼N\left(\;.\;;{\hat{\mu }}_{n},\tilde{\Sigma }\right)\;$$
- draw (u,v) such that
  (29) u∼U([01])andv∼U([01])
- Accept ${\bar{\mu }}_{n+1}$ if and only if
  (30) $$u\leq \min\left(1,\frac{\bar{L}\left({\bar{\mu }}_{n+1};d\right)\pi_{\mu }\left({\bar{\mu }}_{n+1}\right)}{\bar{L}\left({\bar{\mu }}_{n};d\right)\pi_{\mu }\left({\bar{\mu }}_{n}\right)}\right)\;$$
  set ${\bar{\mu }}_{n+1}={\bar{\mu }}_{k}$ otherwise.
- Accept ${\hat{\mu }}_{n+1}$ if and only if
  (31) $$v\leq \min\left(1,\frac{\hat{L}\left({\hat{\mu }}_{n+1};d\right)\pi_{\mu }\left({\hat{\mu }}_{n+1}\right)}{\hat{L}\left({\hat{\mu }}_{n};d\right)\pi_{\mu }\left({\hat{\mu }}_{n}\right)}\right)\;$$
  set ${\hat{\mu }}_{n+1}={\hat{\mu }}_{k}$ otherwise.

The MCMC algorithm is initialised by state $\left({\bar{\mu }}_{0},{\hat{\mu }}_{0}\right)$, where both $({\bar{\mu }}_{0}$ and ${\hat{\mu }}_{0})$ are drawn from distribution $\pi_{0}$.

In the field of a posteriori finite element error estimation, the error estimate is usually a post-processing operation of the coarse finite element solution. This adapts seamlessly to non-Markovian Monte-Carlo samplers, by post-processing each of the independent samples. Here, unfortunately, the finite element model has to be called twice at every iteration of the Markov process. Whether this can be avoided or not, for instance by making use of elements of sequential Monte-Carlo samplers, is unclear to us at this stage.

### 5.3. Machine Learning-Based Simulation of the Discretisation Error

At this stage, any numerical error estimator can be used, provided that it is goal-oriented. A method of choice could be a residual-based [5,6] or smoothing-based a posteriori error estimator [12,46] in conjunction with the adjoint methodology [8,9,11]. In addition, nothing prevents us from using a meta-modelling approach, such as projection-based reduced order modelling [47,48,49,50,51,52] or polynomial chaos expansions [16,17] to approximate the variations of the computed quantities of interest with parameter variation. Error estimates also exist for such two-level approximations.

In this contribution, we develop and use a feature-based method, that finds its roots in data-science methodologies and is much more “black-box” than the previously mentioned strategies. The proposed technique is inspired by the work of [35,36,37].

**Figure 6:**
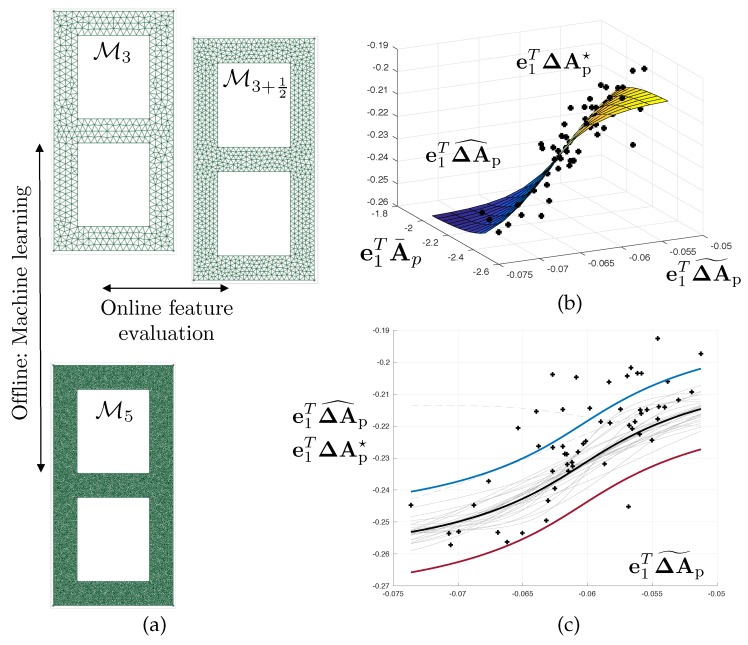
Machine-learning-based error estimation procedure. The overkill error (difference between the current mesh and a much finer mesh as shown in (**a**)) is postulated as being an unknown function of an error estimate. The inexpensive error estimate is the distance between the results obtained when using the current mesh and those obtained when using a slightly refined mesh as shown in. (**b**) The statistical learning is done offline via a Monte-Carlo sampling of the true error (using the prior as sampling density) and the adaptive fitting of a Neural Network regression. The regression is bootstrapped to provide enhanced stability, and derive confidence interval estimates for the overall error estimation procedure which is shown in (**c**). Both the size of the dataset and the hyperparameters of the network are found automatically via a Greedy process that is not detailed in this paper.

The exact continuum mechanics model is well approximated by a very refined numerical strategy (e.g., no meta-modelling and a very fine mesh). However, this very refined numerical model cannot be used at every iteration of MCMC as its evaluation is very costly. We propose to train a model that will map parameter μ to the output of the generally intractable very fine model through the combination of (i) a dedicated feature extractor and (ii) a weakly parametric regression model. This combination is defined as

(32) $$\left(\begin{array}{c} A_{d}^{★}\left(\mu \right)\\ A_{p}^{★}\left(\mu \right) \end{array}\right)-\left(\begin{array}{c} {\bar{A}}_{d}\left(\mu \right)\\ {\bar{A}}_{p}\left(\mu \right) \end{array}\right)\underset{\mathrm{wished}}{=}\left(\begin{array}{c} {\hat{\Delta A}}_{d}\left(\mu \right)\\ {\hat{\Delta A}}_{p}\left(\mu \right) \end{array}\right)\underset{\mathrm{constructed}}{=}R\left(F\left(\mu \right);\theta \right)$$

where the $.^{★}$ denote quantities that are delivered by the overkill (but computable) numerical model and R is a weakly parametrised regression model, here a neural network regression (a Gaussian process could be used as well, but a random forest would probably have been the most efficient choice, given the way we bootstrap the regression model to generate estimates of generalisation errors), parametrised by a set of parameters $\theta \in R^{n_{\theta }}$ (we do not explicitly distinguish parameters and hyper-parameters in our notations). F is a mapping from input μ to a feature space. Its careful design is critical to the success of the machine learning procedure. We choose to construct the following features:

(33) $$F\left(\mu \right)=\left(\begin{array}{c} {\tilde{A}}_{d}\left(\mu \right)-{\bar{A}}_{d}\left(\mu \right)\\ {\bar{A}}_{d}\left(\mu \right)\\ {\tilde{A}}_{p}\left(\mu \right)-{\bar{A}}_{p}\left(\mu \right)\\ {\bar{A}}_{p}\left(\mu \right) \end{array}\right)$$

where ${\tilde{A}}_{d}\left(\mu \right)$ are *slighlty* corrected computational models, here generated by refining the mesh by a moderate factor. In our examples, the typical mesh size is divided by 1.5 (see Figure 2 and Figure 6 where we use mesh $M_{i+\frac{1}{2}}$ to correct mesh $M_{i}$, whilst the overkill solution is computed using $M_{i+2}$). In this fashion, the generation of features will remain of the order of the computation of the finite element solution itself.

Now, we train $n_{d}+n_{p}$ multivariate neural network regression models

(34) $$\left(\begin{array}{c} {\hat{\Delta A}}_{d}\left(\mu \right)\\ {\hat{\Delta A}}_{p}\left(\mu \right) \end{array}\right)=\left(\begin{array}{c} R_{d,1}\left(\left(\begin{array}{c} {e_{1}^{n_{d}}}^{T}\left({\tilde{A}}_{d}\left(\mu \right)-{\bar{A}}_{d}\left(\mu \right)\right)\\ {e_{1}^{n_{d}}}^{T}{\tilde{A}}_{d}\left(\mu \right) \end{array}\right),\theta_{d,1}\right)\\ ...\\ R_{d,n_{d}}\left(\left(\begin{array}{c} {e_{n_{d}}^{n_{d}}}^{T}\left({\tilde{A}}_{d}\left(\mu \right)-{\bar{A}}_{d}\left(\mu \right)\right)\\ {e_{n_{d}}^{n_{d}}}^{T}{\tilde{A}}_{d}\left(\mu \right) \end{array}\right),\theta_{d,n_{d}}\right)\\ R_{p,1}\left(\left(\begin{array}{c} {e_{1}^{n_{p}}}^{T}\left({\tilde{A}}_{p}\left(\mu \right)-{\bar{A}}_{p}\left(\mu \right)\right)\\ {e_{1}^{n_{p}}}^{T}{\tilde{A}}_{p}\left(\mu \right) \end{array}\right),\theta_{p,1}\right)\\ ...\\ R_{p,n_{p}}\left(\left(\begin{array}{c} {e_{n_{p}}^{n_{p}}}^{T}\left({\tilde{A}}_{p}\left(\mu \right)-{\bar{A}}_{p}\left(\mu \right)\right)\\ {e_{n_{p}}^{n_{p}}}^{T}{\tilde{A}}_{p}\left(\mu \right) \end{array}\right),\theta_{p,n_{p}}\right) \end{array}\right)$$

where $e_{j}^{m}$ denotes the jth canonical vector of $R^{m}$.

Each of the regression $R_{l,i}$ is a single-hidden layer bootstrap-aggregated neural network model with $n_{n}$ neurons and $n_{\mathrm{nbs}}$ bootstrap replicates.

(35) $$R_{l,i}\left(\left(\begin{array}{c} x\\ y \end{array}\right),\theta_{l,i}\right)=\frac{1}{k}\sum_{k=1}^{n_{\mathrm{nbs}}}\sum_{j=1}^{n_{n}}a_{j}^{\left(k\right)}\tanh\left(a_{x,j}^{\left(k\right)}x+a_{y,j}^{\left(k\right)}y+o_{j}^{\left(k\right)}\right)+o^{\left(k\right)}$$

#### Training

We sample artificial “data” by running the overkill computational model $n_{\mathrm{ml}}$ times, after sampling the training set parameters $\left\{\mu_{1}^{\mathrm{ml}},\;\mu_{2}^{\mathrm{ml}},\;...\;,\;\mu_{n_{\mathrm{ml}}}^{\mathrm{ml}}\right\}$ from prior $\pi_{\mu }$. The number of neurons and the cardinality of the training set is chosen automatically by making use of an automatised early-stopping methodology that aims to maximise the predictive coefficient of determination. We will not detail this procedure here. Fitting of the nonlinear regression coefficients is performed by employing standard least-squares method, solved by a gradient descent algorithm with randomised initialisation. Outliers of the set of bootstrap replicates are identified and eliminated to decrease the variance of the boostrap-aggregated regression model.

An example of fitted regression model is represented in Figure 6, where the output is the discretisation error in the first free vibration circular frequency (i.e., regression model $R_{d,1}$).

### 5.4. Bootstrap Confidence Intervals for the MCMC Sampler

At any iteration *n* of the MCMC sampler, a Monte-Carlo estimate of the discretisation error for the posterior density of the ith component of μ is given by

(36) $$D_{\mathrm{ks}}^{\left(n\right)}:=D_{\mathrm{ks}}\left({\hat{\pi }}_{\mu_{i}|d}^{\left(n\right)}\left(\mu_{i}\right),{\bar{\pi }}_{\mu_{i}|d}^{\left(n\right)}\left(\mu_{i}\right)\right)=\underset{\mu_{i}}{\sup}\left(\left|({\hat{\Pi }}_{\mu_{i}|d}^{\left(n\right)}\left(\mu_{i}\right)-{\bar{\Pi }}_{\mu_{i}|d}^{\left(n\right)}\left(\mu_{i}\right))\right|\right)\;$$

where

(37) $${\bar{\Pi }}_{\mu_{i}|d=d}^{\left(n\right)}\left(\mu_{i}\right):=\frac{1}{\tilde{n}}\sum_{k=n-\tilde{n}+1}^{n}I\left(\mu_{i};{\bar{\mu }}_{k,i}\leq \mu_{i}\right)\;,$$

and

(38) $${\hat{\Pi }}_{\mu_{i}|d=d}^{\left(n\right)}\left(\mu_{i}\right):=\frac{1}{\tilde{n}}\sum_{k=n-\tilde{n}+1}^{n}I\left(\mu_{i};{\hat{\mu }}_{k,i}\leq \mu_{i}\right).$$

Crucially, $D_{\mathrm{ks}}^{\left(n\right)}$ is a random variable whose statistics, and in particular its bias and variance, strongly depend on the length of the Markov chain. Unfortunately, evaluating the convergence of any statistics provided by MCMC is difficult, due to the statistical dependency between successively drawn samples.

**Figure 7:**
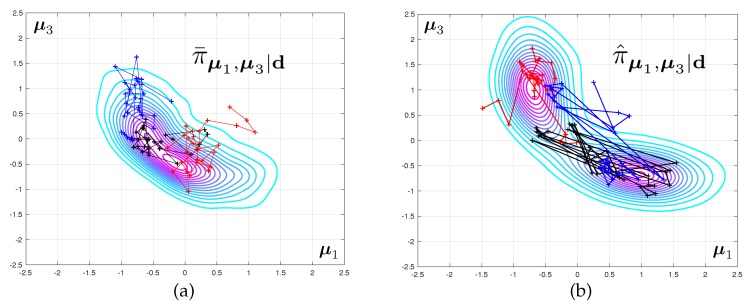
Multiple MCMC routines were run in parallel, for both the coarse likelihood function (coarse mesh) as shown in (**a**) and the enhanced likelihood function obtained after correction by the goal-oriented finite element error estimate, as shown in (**b**). The results are pooled, and will be boostrapped to derive confidence intervals for the summary statistics of the posterior densities.

Following standard diagnostic approaches for MCMC samplers (e.g., the Gelman-Rubin convergence test [53,54]), we will run $n_{c}\geq 10$ independent (tempered) MCMC chains in parallel and pool all the resulting samples, after discarding the first 25% of every individual chain as burn-in (see Figure 7 as a visual aid). The pooled samples at iteration *n* of the multiple-chain MCMC (MC${}^{3}$) algorithm are

(39) $${\bar{S}}^{\left(n\right)}=\prod_{i=1}^{n_{c}}{\bar{S}}_{i}^{\left(n\right)}\;{\hat{S}}^{\left(n\right)}=\prod_{i=1}^{n_{c}}\;{\hat{S}}_{i}^{\left(n\right)}$$

where ∏ denotes the cartesian product. In the previous expression, the sample set from chain *i* (at ambient temperature) is

(40) $${\bar{S}}_{i}^{\left(n\right)}=\left\{{\bar{\mu }}_{i,n-\tilde{n}+1},\;\cdots \;,{\bar{\mu }}_{i,n}\right\}\;{\hat{S}}_{i}^{\left(n\right)}=\left\{{\hat{\mu }}_{i,n-\tilde{n}+1},\;\cdots \;,{\hat{\mu }}_{i,n}\right\}$$

$D_{\mathrm{ks}}^{\left(n\right)}$ is now the pooled KS distance estimate provided by the MC${}^{3}$ algorithm (we will keep the same notation for the sake of simplicity). Formally, we simply replace Equation (37) by

(41) $${\bar{\Pi }}_{\mu_{i}|d=d}^{\left(n\right)}\left(\mu_{i}\right):=\frac{1}{n_{c}}\frac{1}{\tilde{n}}\sum_{l=1}^{n_{c}}\sum_{k=n-\tilde{n}+1}^{n}I\left(\mu_{i};{\bar{\mu }}_{l,k,i}\leq \mu_{i}\right),$$

and perform a similar operation to define the pooled corrected empirical distribution.

The independence of the $n_{c}$ MCMC chains allows us to compute confidence intervals for $D_{\mathrm{ks}}^{\left(n\right)}$ by making use of the non-parametric bootstrap. This is done by resampling ${\bar{S}}^{\left(n\right)}$ and ${\hat{S}}^{\left(n\right)}$ with replacement, generating bootstrap replicates of the pooled sample sets ${\left\{{\bar{S}}_{k}^{\left(n\right)}\right\}}_{k\in 〚1\;n_{\mathrm{bs}}〛}$ and ${\left\{{\hat{S}}_{k}^{\left(n\right)}\right\}}_{k\in 〚1\;n_{\mathrm{bs}}〛}$,

(42) $${\bar{S}}_{k}^{\left(n\right)}=\prod_{i=\in B_{k}}{\bar{S}}_{i}^{\left(n\right)}\;{\hat{S}}_{k}^{\left(n\right)}=\prod_{i=\in B_{k}}{\hat{S}}_{i}^{\left(n\right)}\;,$$

where $B_{k}\in {\left(〚1\;n_{c}〛\right)}^{n_{\mathrm{bs}}}$ is such that each element of this set is drawn uniformly over $〚1\;n_{c}〛$ and *k* varies between 1 and a large number $n_{\mathrm{bs}}$, typically set to 1000. For each replicate, statistics $D_{\mathrm{ks}}^{\left(n\right)}$, denoted by $D_{\mathrm{ks},k}^{\left(n\right)}$, can be computed in a straightforward manner by using the bootstrap replicates of the pooled empirical distributions

(43) $${\bar{\Pi }}_{\mu_{i}|d=d,k}^{\left(n\right)}\left(\mu_{i}\right):=\frac{1}{n_{c}}\frac{1}{\tilde{n}}\sum_{{\bar{\mu }}_{i}^{★}\in {\bar{S}}_{k}^{\left(n\right)}}I\left(\mu_{i};{\bar{\mu }}_{i}^{★}\leq \mu_{i}\right)\;,$$

(44) $${\hat{\Pi }}_{\mu_{i}|d=d,k}^{\left(n\right)}\left(\mu_{i}\right):=\frac{1}{n_{c}}\frac{1}{\tilde{n}}\sum_{{\hat{\mu }}_{i}^{★}\in {\hat{S}}_{k}^{\left(n\right)}}I\left(\mu_{i};{\hat{\mu }}_{i}^{★}\leq \mu_{i}\right)\;,$$

which reads as

(45) $$D_{\mathrm{ks},k}^{\left(n\right)}:=D_{\mathrm{ks}}\left({\hat{\pi }}_{\mu_{i}|d,k}^{\left(n\right)}\left(\mu_{i}\right),{\bar{\pi }}_{\mu_{i}|d,k}^{\left(n\right)}\left(\mu_{i}\right)\right)=\underset{\mu_{i}}{\sup}\left(\left|({\hat{\Pi }}_{\mu_{i}|d,k}^{\left(n\right)}\left(\mu_{i}\right)-{\bar{\Pi }}_{\mu_{i}|d,k}^{\left(n\right)}\left(\mu_{i}\right))\right|\right),$$

Finally, the bootstrap confidence intervals are calculated by calculating the *X*th and (100−X)th bootstrap percentiles such that the *X*th percentile reads as

(46) $$q_{X}^{\left(n\right)}=\left(Q_{X}\left({\left\{D_{\mathrm{ks},k}^{\left(n\right)}\right\}}_{k\in 〚1\;n_{\mathrm{bs}}〛}\right)-\mathrm{median}\left({\left\{D_{\mathrm{ks},k}^{\left(n\right)}\right\}}_{k\in 〚1\;n_{\mathrm{bs}}〛}\right)\right)+D_{\mathrm{ks}}^{\left(n\right)}$$

where $Q_{X}$ is an operator that extracts the *X*th and *Y*th percentile of the set passed as argument.

It is important to understand that the derived bootstrap confidence interval stands for a chain of finite length *n*, and not for the asymptotic limit. In fact, estimate $D_{\mathrm{ks}}^{\left(n\right)}$ of $D_{\mathrm{ks}}$ is strongly biased (upward for small asymptotic errors $D_{\mathrm{ks}}$ and typically downward for large asymptotic errors), which is due to two factors:

- the existence of the burn-in phase. For small *n*, each individual chain will be strongly affected by the initialisation of the chains. For small asymptotic errors, this can be expected to have a strong upward bias effect providing that the initialisation is disperse, which is often the case in practice (e.g., initialisation from the prior, sequential MC approaches with decreasing levels of noise). This can be visualised in Figure 7. Two incomplete chains running on the same probability density may be exploring completely different regions of space, yielding values of $D_{\mathrm{ks}}$ that are large, even in the case where the corrected and uncorrected densities are close to one another.
- the discrete evaluation of the KS distance itself, which generates an additional (upward) bias.

The variance of $D_{\mathrm{ks}}^{\left(n\right)}$ decreases with the number of chains of the MC${}^{3}$ algorithm (which is not a free parameter as overall CPU cost increases linearly with $n_{c}$). Of course, bias and variance are both expected to decrease with the length *n* of the run.

### 5.5. Simultaneous Control of All Sources of Errors

We now make use of the CI derived for $D_{\mathrm{ks}}^{\left(n\right)}$ to construct an adaptive inverse problem solver that jointly controls the quality of the mesh, and that of the statistical evaluation of the posterior densities. The algorithm is as follows. Given a current mesh $M_{i}$ and number of iterations *n* of the Monte-Carlo solver, do:

- If n=0, perform $m_{0}$ iterations of the MC${}^{3}$ algorithm and set $n\leftarrow n+m_{0}$.
- Evaluate mesh convergence criterion $C_{m}$. If this criterion is satisfied, exit the adaptation procedure;
- If $C_{m}$ is not satisfied, evaluate statistical convergence criterion $C_{s}$
  - If $C_{s}$ is satisfied, set i←i+1, reinitialise the MC${}^{3}$ algorithm and set n=0;
  - otherwise perform m(n) iterations of the MC${}^{3}$ algorithm and set n←n+m(n) (*m* should be an exponentially increasing function).

#### 5.5.1. Criterion $C_{m}$

Convergence of the finite element-based Bayesian inverse problem is achieved when $D_{\mathrm{ks}}\leq \gamma$, where γ is a numerical tolerance that will typically be chosen between 0.01 and 0.2. As we only have Monte-Carlo estimates of $D_{\mathrm{ks}}$, we require instead that

(47) $$C_{m}:\;q_{100-X}^{\left(n\right)}\leq \gamma$$

Criterion $C_{m}$ is an indicator of the combined effect of the discretisation and statistical errors onto the posterior densities. The discretisation error is evaluated through the choice of measure $D_{\mathrm{ks}}$ as reliability indicator, whilst the statistical error is taken into account by making use of the upper limit of the bootstrap confidence interval for $D_{\mathrm{ks}}^{\left(n\right)}$. The risk of falsely detecting mesh convergence due to a high statistical error is small, due to the fact that $D_{\mathrm{ks}}^{\left(n\right)}$ is an upwardly biased estimate of $D_{\mathrm{ks}}$ for small $D_{\mathrm{ks}}$. Confidence in the result can be increased through a loose Gelman-Rubin convergence test in order to eliminate the risk of stopping the MC${}^{3}$ algorithm in its non-ergodic phase. However, this has proved to be unnecessary in our numerical tests. This is because $C_{m}$ is a rather strict criterion.

#### 5.5.2. Criterion $C_{s}$

The second criterion will help us determine whether mesh refinement is actually needed, or whether the statistical error is too large for use to take a robust decision regarding mesh refinement. The ideal criterion is $D_{\mathrm{ks}}\geq \eta$, where η<γ. The obvious strategy that would require $q_{X}^{\left(n\right)}\leq \eta$ does not work. This is due to the previously explained upward bias of estimator $D_{\mathrm{ks}}^{\left(n\right)}$, which would eventually lead to the spurious satisfaction $C_{s}$, and consequently to systematic mesh refinement operations for low *n* count even when the mesh becomes fine enough for $D_{\mathrm{ks}}$ to be well below target γ. In order to derive an appropriate criterion $C_{s}$, we remark that whilst the ideal criterion $D_{\mathrm{ks}}\geq \eta$ cannot be statistically evaluated for general values of η, criterion $D_{\mathrm{ks}}>0$ can be. More precisely, we propose to estimate the statistical error by evaluation whether, at current *n*, the corrected and uncorrected posterior distributions can be statistically distinguished.

We postulate the following null hypothesis: Hypothesis 0 (H0): *the corrected and uncorrected posterior densities are identical*.

The rejection of the null hypothesis will indicate that the two densities are significantly different. Now, the criterion for the need for mesh refinement becomes the following:

(48) $$C_{s}:\;\mathrm{Pr}\left(D_{\mathrm{ks}}^{\left(n\right)}\geq q_{Z}^{\left(n\right)}|H0\right)\leq \xi$$

The probability density of $D_{\mathrm{ks}}^{\left(n\right)}$ under null hypothesis H0 can be approximated by adapting the previously described bootstrap procedure. We will estimate the density of

(49) $$D_{\mathrm{ks},0}^{\left(n\right)}:=D_{\mathrm{ks}}\left({\bar{\pi }}_{\mu_{i}|d}^{(n),1}\left(\mu_{i}\right),{\bar{\pi }}_{\mu_{i}|d}^{(n),2}\left(\mu_{i}\right)\right)=\underset{\mu_{i}}{\sup}\left(\left|({\bar{\Pi }}_{\mu_{i}|d}^{(n),1}\left(\mu_{i}\right)-{\bar{\Pi }}_{\mu_{i}|d}^{(n),2}\left(\mu_{i}\right))\right|\right)\;$$

where ${\bar{\pi }}_{\mu_{i}|d}^{(n),1}$ and ${\bar{\pi }}_{\mu_{i}|d}^{(n),2}$ are obtained, respectively, by pooling the result of two independent runs of $n_{c}$ Markov chains of lengths *n* and corresponding to the uncorrected finite element model only (one can equally choose the corrected one). Notice, to clarify the idea, that $D_{\mathrm{ks},0}^{\left(n\right)}$ tends to 0 as *n* tends to infinity: this is a measure of the statistical error only. The desired density can be estimated by resampling ${\bar{S}}^{\left(n\right)}$ twice, computing the KS distance between the two pooled sets of samples, and repeating the operation $n_{\mathrm{bs}}$ times, which generates a sequence of real numbers $\left\{D_{\mathrm{ks},0,k}^{\left(n\right)}\right\}$. The replicated empirical cumulative distributions are

(50) $${\bar{\Pi }}_{\mu_{i}|d=d,k}^{(n),j}\left(\mu_{i}\right):=\frac{1}{n_{c}}\frac{1}{\tilde{n}}\sum_{{\bar{\mu }}_{i}^{★}\in {\bar{S}}_{k}^{(n),j}}I\left(\mu_{i};{\bar{\mu }}_{i}^{★}\leq \mu_{i}\right)\;$$

with the sampling sets

(51) $${\bar{S}}_{k}^{(n),j}=\prod_{i=\in B_{k}^{j}}{\bar{S}}_{i}^{\left(n\right)}\;$$

where j=1 or j=2 and $B_{k}^{1}$ and $B_{k}^{2}$ are sets of $〚1\;n_{c}{〛}^{n_{c}}$ constructed such that each element of these two sets is drawn uniformly over $〚1\;n_{c}〛$. We can now extract the (100×(1−ξ))th percentile $q_{Y,0}^{\left(n\right)}$ corresponding to *p*-value ξ, and evaluate whether $q_{Y,0}^{\left(n\right)}\leq q_{Y}^{\left(n\right)}$. If so, test $C_{s}$ is true. If not, the statistical sampling error is too large to allow us to decide whether mesh refinement should be performed or not. In this case, and assuming that mesh convergence criterion $C_{m}$ is not satisfied, we need to continue sampling with MCMC.

Notice that the use of a larger percentile *Z* yields a less conservative indicator for the need for mesh refinement, which can be compensated by decreasing *p*-value ξ. The criterion that is arguably the easiest to interpret is $\mathrm{Pr}\left(D_{\mathrm{ks}}^{\left(n\right)}\geq q_{50}^{\left(n\right)}|H0\right)\leq \xi$, where $q_{50}^{\left(n\right)}$ is directly the KS distance $D_{\mathrm{ks}}^{\left(n\right)}$ (i.e., computed without bootstrapping).

## 6. Numerical Examples—Part II. Automatised Error Control and Discussion

We now come back to the numerical examples introduced in Section 3 and produce three series of results, illustrating three difference aspects of the proposed approach.

### 6.1. MCMC Iterations Only When Needed

**Figure 8:**
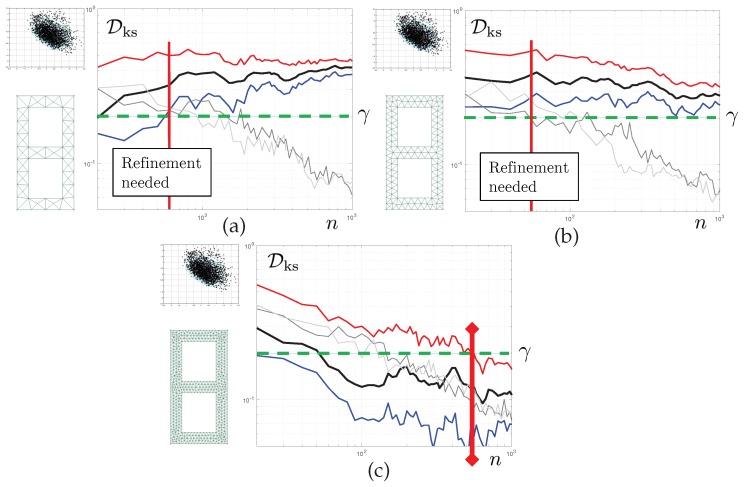
Convergence of the KS distance as a function of the number of Monte-Carlo samples in each chain (black line, with confidence interval represented by the area between the blue and red lines). The mesh used is represented schematically on the left-hand side of each of the graphs in (**a**–**c**). The data is composed of a unique noisy measurement of the first eigenfrequency. The quantity of interest is here the first elasticity parameter, in log scale. The grey lines are an indicator of the bias of the KS distance estimate, which decreases with the sample size. The bias should be be small for the estimate to be robust, but not too small so as to avoid running unnecessarily long chains using meshes that are too coarse for our needs. Sufficiently accurate results are obtained with the third mesh. Importantly, very few MCMC iterations are needed to determine that the two first meshes are too coarse for our needs.

The results reported in Figure 8 correspond to Test 1. For now, we aim to control the quality of the posterior density of the first parameter $\mu_{1}$. Each of the graphs shows the evolution of various statistics of the corresponding KS distance as a function of the number of iteration *n* of the tempered MC${}^{3}$ algorithm. The mesh used is displayed on the left-hand side of each of the graphs. The solid black line represents the evolution $D_{\mathrm{ks}}^{\left(n\right)}$ itself. The 5th and and 95th bootstrap percentiles of this quantity are also reported, in solid blue line and solid red line, respectively. We set convergence criterion $C_{m}$ such that γ=0.2 and Y=95 for the pure discretisation error. Therefore, convergence with respect to the finite element discretisation is obtained when, for sufficient long chains, the red line (upper limit of the bootstrap CI for $D_{\mathrm{ks}}^{\left(n\right)}$) is below γ.

In grey colours, we report evolution of $D_{\mathrm{ks},0}^{\left(n\right)}$ for the coarse scale (light grey), consistently with the derivations of the previous section, but also, for reference, a similar statistics constructed with the corrected finite element model (dark grey). Both curves have similar evolutions and, of course, tend to 0 as n tends to infinity. We set Z=5, and η=0.05. This means that the statistical error is considered small enough once the blue line (lower limit of the bootstrap CI for $D_{\mathrm{ks}}^{\left(n\right)}$) is above the 95th percentile of $D_{\mathrm{ks},0}^{\left(n\right)}$. Here, “small enough” is to be understood, as small enough for a decision regarding the need to refine the mesh one step further to be taken.

The results are as follows. For the coarsest mesh, we detect a separation of the discretisation and statistical errors at iteration 60 (vertical solid red line). $C_{s}$ is satisfied. As $C_{m}$ is not satisfied, we can stop the MCMC sampler and refine the mesh. A similar behaviour is seen for mesh $M_{2}$. For the last mesh, convergence is achieved after 530 iterations, $C_{s}$ never reaching satisfaction. The discretisation and sampling errors remain entangled, but the sum of them is below the desired target.

We can see here that the proposed algorithm allows us to stop the MCMC sampler early, moving directly to the level of mesh refinement that will yield the desired quality of the posterior densities.

### 6.2. Goal-Oriented Error Control

**Figure 9:**
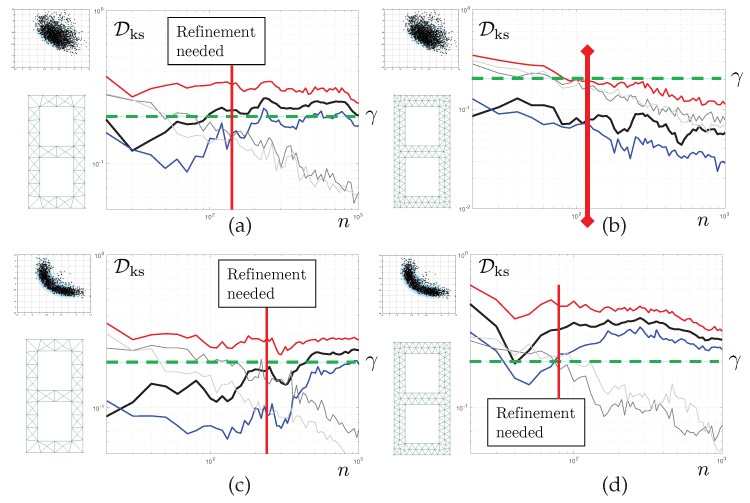
Convergence of the KS distance as a function of the number of Monte-Carlo samples in each chain. The (**a**,**b**) correspond to the inverse problem where only the first eigenfrequency is observed. (**c**,**d**) corresponds to the case where the first three eigenfrequencies are observed. The quantity of interest is the fourth eigenvalue, which is unobserved in both cases and must be inferred. The corresponding posterior density is illustrated above the mesh pictogram. Interestingly, the data-poor problem (**a**,**b**) can be solved appropriately with a coarse mesh, while the data-rich case (**c**,**d**) requires a much finer grid to be solved accurately.

The second set of results correspond to Test 1, still. However, we now monitor the convergence of the posterior predictive density of the fourth eigenvalue. This is reported in the top two graphs of Figure 9. For $M_{1}$ Early-stopping is performed at iteration 40 of the MCMC sampler. For $M_{2}$, global convergence is achieved after 120 iterations, and this error keeps decreasing. The MCMC is allowed to continue generating samples, meaning that the discretisation error is actually a lot smaller than the requested tolerance γ. It is interesting to see that the convergence is much faster than for the first parameter, whose convergence was studied in the previous subsection. This shows that for the same inverse problem, the algorithm may spend more or less resources depending on the engineering quantity of interest.

### 6.3. Uncertainty-Driven Error Control

Finally, the last set of results concerns Test 2. Here, remember that the three first eigenvalues are used as measurements. We control the convergence of the fourth eigenvalue, which was controlled in the context of Test 1 in the previous subsection. We see here that for mesh $M_{2}$, the posterior density of the fourth eigenvalue is still far from convergence, whilst it was evaluated in a very precise way in Test 1. This shows that the proposed error control algorithm automatically adapts to the level of posterior uncertainty. Qualitatively, a wide posterior density is associated with a high uncertainty concerning the value of QoIs. Consequently, the mesh does not need to be very refined to capture the posterior density correctly. Conversely, for Test 2, the posterior density is sharper, and similar levels of discretisation errors have a much stronger impact on the KS distance.

## 7. Concluding Remarks and Discussion

We have presented a methodology to control the various sources of errors arising in finite-element based Bayesian inverse problems. We have focussed on a simple numerical approximation chain consisting of (i) a finite element discretisation of the continuum mechanics problem and (ii) a MCMC solver to draw samples from posterior density distributions. So far we did not consider further error sources such as that engendered by meta-modelling. We have showed that it was possible to drive the mesh refinement process in a goal-oriented manner, by quantifying the impact of the associated error onto posterior density distributions. In order to do so, we run two independent MCMC simultaneously, one of them using a corrected likelihood function that takes the discretisation error. Any a posteriori error estimate available for the PDE under consideration may be used to obtain this correction. Of course, the accuracy (effectivity) of the chosen error estimate will impact the accuracy of the methodology developed in this paper. The study and control of this effect will require further research to be carried out,

We have shown that by using multiple replicates of the component-wise MCMC, it was also possible to (i) derive bootstrap-based confidence intervals for the error in posterior densities engendered by the spatial discretisation of the underlying PDE. Importantly, this approach allows us to stop the MCMC iteration as soon as we can be sufficiently confident in the fact that the posterior densities obtained with the current mesh are not accurate enough for our purpose. The approach is goal-oriented: the adaptivity is performed in the sense of the posterior distribution of either some of the latent parameters, or in the sense of the predictive posterior density of engineering QoIs. Finally, we have shown that the approach is uncertainty driven: it will only refine the mesh and/or request additional samples to be drawn by the MCMC algorithm if the effect on the QoIs can be felt when measured using a statistical distance between their posterior densities. In particular, model updating problems with wider priors tend to require less computational effort than parameter estimation problems with very rich observed data and/or narrow prior densities. The proposed methodology establishes a bridge between the field of reliability estimation for the sampling-based algorithms used to solve probabilistic inverse problems, and the field of deterministic, goal-oriented finite element error estimation.

Although the results presented in this paper are encouraging, the proposed approach has shortcomings that need to be addressed in future research work. The error estimation procedure is relatively wasteful for two reasons. Firstly, multiple MCMC algorithms need to run independently for bootstrapping to be possible. These chains all exhibit their own burn-in phase, which increases the amount of discarded samples. Secondly, the finite element error estimation procedure is not merely a post-processing operation any longer; one cannot post-process the finite element results corresponding to the uncorrected chain in order to compute the corrected likelihood. This is because both chains are independent and require running the coarse finite element simulations at different points of the parameter domain. Finally, let us acknowledge that Bayesian finite element inverse problems should not be solved by a MCMC solver without constructing a surrogate model first. The number of finite element computations involved, even if the proposal distribution is well designed, is in the tens of thousands. We are currently investigating the use of Polynomial Chaos surrogates, which adds another layer of numerical approximation that needs to be controlled in a robust and efficient manner. In this context, an elegant approach to separate the sources of errors (i.e., Finite Element discretisation error and Polynomial Chaos error) may be found in [55], which may constitute a solid starting point for the next step of our investigations.

## References

[1] Nagel J.B., Sudret B. (2016). A Unified Framework for Multilevel Uncertainty Quantification in Bayesian Inverse Problems. Probab. Eng. Mech..

[2] Beck J.L., Au S.K. (2002). Bayesian Updating of Structural Models and Reliability Using Markov Chain Monte Carlo Simulation. J. Eng. Mech..

[3] Chen P., Schwab C. (2016). Sparse-Grid, Reduced-Basis Bayesian Inversion: Nonaffine-Parametric Nonlinear Equations. J. Comput. Phys..

[4] Cotter S.L., Dashti M., Stuart A.M. (2010). Approximation of Bayesian Inverse Problems for PDEs. SIAM J. Numer. Anal..

[5] Ainsworth M., Oden J. (2000). A Posteriori Error Estimation in Finite Element Analysis.

[6] Ladevèze P., Pelle J.P. (2004). Mastering Calculations in Linear and Non Linear Mechanics.

[7] Díez P., Parés N., Huerta A. (2010). Encyclopedia of Aerospace Engineering.

[8] Oden J.T., Prudhomme S., Oden J.T., Prudhomme S. (1999). Goal-Oriented Error Estimation and Adaptivity for the Finite Element Method. Comput. Methods Appl. Mech. Eng..

[9] Cirak F., Ramm E. (2000). A Posteriori Error Estimation and Adaptivity for Elastoplasticity Using the Reciprocal Theorem. Int. J. Numer. Methods Eng..

[10] Strouboulis T., Babuŝka I., Datta D.K., Copps K., Gangaraj S.K. (2000). A Posteriori Estimation and Adaptive Control of the Error in the Quantity of Interest. Part I: A Posteriori Estimation of the Error in the von Mises Stress and the Stress Intensity Factor. Comput. Methods Appl. Mech. Eng..

[11] Becker R., Rannacher R. (2001). An Optimal Control Approach to a Posteriori Error Estimation in Finite Element Methods. Acta Numer..

[12] González-Estrada O., Nadal E., Ródenas J., Kerfriden P., Bordas S.A., Fuenmayor F. (2013). Mesh Adaptivity Driven by Goal-Oriented Locally Equilibrated Superconvergent Patch Recovery. Comput. Mech..

[13] Jensen H., Esse C., Araya V., Papadimitriou C. (2017). Implementation of an Adaptive Meta-Model for Bayesian Finite Element Model Updating in Time Domain. Reliab. Eng. Syst. Saf..

[14] Au S.K., Zhang F.L. (2016). Fundamental Two-Stage Formulation for Bayesian System Identification, Part I: General Theory. Mech. Syst. Signal Process..

[15] Zhang F.L., Au S.K. (2016). Fundamental Two-Stage Formulation for Bayesian System Identification, Part II: Application to Ambient Vibration Data. Mech. Syst. Signal Process..

[16] Babǔska I., Tempone R., Zouraris G.E. (2004). Galerkin Finite Element Approximations of Stochastic Elliptic Partial Differential Equations. SIAM J. Numer. Anal..

[17] Ghanem R.G., Spanos P.D. (2003). Stochastic Finite Elements: A Spectral Approach.

[18] Nouy A. (2009). Recent Developments in Spectral Stochastic Methods for the Numerical Solution Ofstochastic Partial Differential Equations. Arch. Comput. Methods Eng..

[19] Kundu A., DiazDelaO F., Adhikari S., Friswell M. (2014). A Hybrid Spectral and Metamodeling Approach for the Stochastic Finite Element Analysis of Structural Dynamic Systems. Comput. Methods Appl. Mech. Eng..

[20] Ganapathysubramanian B., Zabaras N. (2007). Sparse Grid Collocation Schemes for Stochastic Natural Convection Problems. J. Comput. Phys..

[21] Foo J., Karniadakis G.E. (2010). Multi-Element Probabilistic Collocation Method in High Dimensions. J. Comput. Phys..

[22] Pradlwarter H.J., Schuëller G.I. (1997). On Advanced Monte Carlo Simulation Procedures in Stochastic Structural Dynamics. Int. J. Non-Linear Mech..

[23] Yamazaki F., Shinozuka M. (1988). Digital Generation of Non-Gaussian Stochastic Fields. J. Eng. Mech..

[24] Au S.K., Beck J.L. (1999). A New Adaptive Importance Sampling Scheme for Reliability Calculations. Struct. Saf..

[25] Rosenblueth E. (1975). Point Estimates for Probability Moments. Proc. Natl. Acad. Sci. USA.

[26] Christian J.T., Baecher G.B. (2002). The Point-estimate Method with Large Numbers of Variables. Int. J. Numer. Anal. Methods Geomech..

[27] Julier S.J. The Scaled Unscented Transformation. Proceedings of the 2002 American Control Conference.

[28] Yuen K.V., Kuok S.C. (2011). Bayesian Methods for Updating Dynamic Models. Appl. Mech. Rev..

[29] Cui T., Marzouk Y., Willcox K. (2016). Scalable Posterior Approximations for Large-Scale Bayesian Inverse Problems via Likelihood-Informed Parameter and State Reduction. J. Comput. Phys..

[30] Kundu A., Matthies H.G., Friswell M.I. (2018). Probabilistic optimization of engineering system with prescribed target design in a reduced parameter space. Comput. Methods Appl. Mech. Eng..

[31] Schillings C., Schwab C. (2013). Sparse, Adaptive Smolyak Quadratures for Bayesian Inverse Problems. Inverse Probl..

[32] Chen P., Schwab C., Garcke J., Pflüger D. (2016). Adaptive Sparse Grid Model Order Reduction for Fast Bayesian Estimation and Inversion. Sparse Grids and Applications—Stuttgart 2014.

[33] Mattis S.A., Wohlmuth B. (2018). Goal-Oriented Adaptive Surrogate Construction for Stochastic Inversion. Comput. Methods Appl. Mech. Eng..

[34] Pares N., Diez P., Huerta A. (2006). Subdomain-Based Flux-Free a Posteriori Error Estimators. Comput. Methods Appl. Mech. Eng..

[35] Drohmann M., Carlberg K. (2015). The ROMES Method for Statistical Modeling of Reduced-Order-Model Error. SIAM/ASA J. Uncertain. Quantif..

[36] Paul-Dubois-Taine A., Amsallem D. (2014). An Adaptive and Efficient Greedy Procedure for the Optimal Training of Parametric Reduced-Order Models. Int. J. Numer. Methods Eng..

[37] Goury O., Amsallem D., Bordas S.P.A., Liu W.K., Kerfriden P. (2016). Automatised Selection of Load Paths to Construct Reduced-Order Models in Computational Damage Micromechanics: From Dissipation-Driven Random Selection to Bayesian Optimization. Comput. Mech..

[38] Trehan S., Carlberg K.T., Durlofsky L.J. (2017). Error Modeling for Surrogates of Dynamical Systems Using Machine Learning. Int. J. Numer. Methods Eng..

[39] Ciarlet P.G. (1978). The Finite Element Method for Elliptic Problems.

[40] Haario H., Laine M., Mira A., Saksman E. (2006). DRAM: Efficient Adaptive MCMC. Stat. Comput..

[41] Andrieu C., Thoms J. (2008). A Tutorial on Adaptive MCMC. Stat. Comput..

[42] Cheung S.H., Beck J.L. (2009). Bayesian Model Updating Using Hybrid Monte Carlo Simulation with Application to Structural Dynamic Models with Many Uncertain Parameters. J. Eng. Mech..

[43] Geyer C.J. (1991). Markov Chain Monte Carlo Maximum Likelihood.

[44] Neal R.M. (1996). Sampling from Multimodal Distributions Using Tempered Transitions. Stat. Comput..

[45] Lam H.F., Yang J.H., Au S.K. (2018). Markov Chain Monte Carlo-based Bayesian Method for Structural Model Updating and Damage Detection. Struct. Control Health Monit..

[46] Zienkiewicz O.C., Zhu J.Z. (1987). A Simple Error Estimator and Adaptive Procedure for Practical Engineerng Analysis. Int. J. Numer. Methods Eng..

[47] Prud’homme C., Rovas D.V., Veroy K., Machiels L., Maday Y., Patera A.T., Turinici G. (2002). Reliable Real-Time Solution of Parametrized Partial Differential Equations: Reduced-Basis Output Bound Methods. J. Fluids Eng..

[48] Ryckelynck D., Benziane D.M. (2010). Multi-Level a Priori Hyper-Reduction of Mechanical Models Involving Internal Variables. Comput. Methods Appl. Mech. Eng..

[49] Carlberg K., Farhat C., Cortial J., Amsallem D. (2013). The GNAT Method for Nonlinear Model Reduction: Effective Implementation and Application to Computational Fluid Dynamics and Turbulent Flows. J. Comput. Phys..

[50] Kerfriden P., Ródenas J.J., Bordas S.P.A. (2014). Certification of Projection-Based Reduced Order Modelling in Computational Homogenisation by the Constitutive Relation Error. Int. J. Numer. Methods Eng..

[51] Cui T., Marzouk Y.M., Willcox K.E. (2015). Data-Driven Model Reduction for the Bayesian Solution of Inverse Problems. Int. J. Numer. Methods Eng..

[52] Hoang K., Kerfriden P., Bordas S. (2016). A Fast, Certified and “Tuning Free” Two-Field Reduced Basis Method for the Metamodelling of Affinely-Parametrised Elasticity Problems. Comput. Methods Appl. Mech. Eng..

[53] Gelman A., Rubin D.B. (1992). Inference from Iterative Simulation Using Multiple Sequences. Stat. Sci..

[54] Brooks S.P., Gelman A. (1998). General Methods for Monitoring Convergence of Iterative Simulations. J. Comput. Graph. Stat..

[55] Chamoin L., Florentin E., Pavot S., Visseq V. (2012). Robust Goal-Oriented Error Estimation Based on the Constitutive Relation Error for Stochastic Problems. Comput. Struct..

